# Synergy of Oxygen Vacancy and Surface Modulation Endows Hollow Hydrangea-like MnCo_2_O_4.5_ with Enhanced Capacitive Performance

**DOI:** 10.3390/ijms25105075

**Published:** 2024-05-07

**Authors:** Gaofeng Li, Yanyan Li, Pengfei Wang, Lingling Chen, Longfei Li, Chen Bao, Jianfei Tu, Dianbo Ruan

**Affiliations:** 1 Faculty of Mechanical Engineering and Mechanics, Institute of Advanced Energy Storage Technology and Equipment, Ningbo University, Ningbo 315211, China; 2 Institute of Advanced Energy Storage Technology and Equipment, School of Materials Science and Chemical Engineering, Ningbo University, Ningbo 315211, China

**Keywords:** hollow structure, vacancy, nanosheet, supercapacitors

## Abstract

Surface chemistry and bulk structure jointly play crucial roles in achieving high-performance supercapacitors. Here, the synergistic effect of surface chemistry properties (vacancy and phosphorization) and structure-derived properties (hollow hydrangea-like structure) on energy storage is explored by the surface treatment and architecture design of the nanostructures. The theoretical calculations and experiments prove that surface chemistry modulation is capable of improving electronic conductivity and electrolyte wettability. The structural engineering of both hollow and nanosheets produces a high specific surface area and an abundant pore structure, which is favorable in exposing more active sites and shortens the ion diffusion distance. Benefiting from its admirable physicochemical properties, the surface phosphorylated MnCo_2_O_4.5_ hollow hydrangea-like structure (P-MnCoO) delivers a high capacitance of 425 F g^−1^ at 1 A g^−1^, a superior capability rate of 63.9%, capacitance retention at 10 A g^−1^, and extremely long cyclic stability (91.1% after 10,000 cycles). The fabricated P-MnCoO/AC asymmetric supercapacitor achieved superior energy and power density. This work opens a new avenue to further improve the electrochemical performance of metal oxides for supercapacitors.

## 1. Introduction

According to the charge storage mechanism, electrode materials of supercapacitors are mainly classified into two types: electric double-layer materials and pseudocapacitive materials. The former carbonaceous-based materials are typically representative and store electrical energy via ion adsorption/desorption on the electrode surface [1]. The latter makes use of surface or near-surface redox reactions to store energy, such as metal oxides, conducting polymers, and metal sulfides [2]. Therefore, the surface chemistry properties of electrode materials play a significant role in storage capacity and rate capability by influencing the reaction activity and kinetics. On the other hand, structural properties of electrode materials have a great effect on cycling life because repeated stress can affect structural integrity during the continuous charging/discharging process. Thus, the structural and surface properties together determine the electrochemical properties of supercapacitors.

Transition metal oxides (TMOs) have undergone extensive research and application in energy storage, such as supercapacitors and Li-ion batteries [3]. However, TMOs are not sufficient to meet growing energy storage requirements due to low specific capacitance and inferior rate performance. It is generally known that the sluggish reaction kinetics and low utilization/activity of TMOs as active materials are the root causes of their poor performance. In view of the above two crucial influencing factors, researchers have made great progress in the development and optimization of promising TMO electrodes. In various technologies, surface engineering is regarded as the most direct and effective way to improve reaction kinetics and electrochemical activity, including surface modification, coating, and doping. For example, Liu and co-workers verified that introducing oxygen vacancies on the CuCo_2_O_4_ surface through thermal treatment in a hypoxic atmosphere can increase the electrical conductivity and hydrophilic properties, which is beneficial to gain high-rate performance [4]. Cheng et al. constructed the oxygen vacancies and P-doped NiMoO_4_ interface through the combination of phosphorization and N_2_ plasma treatment, which efficiently increased the electrochemically active sites, intrinsic conductivity and facilitated the surface wettability and electrolyte permeation [5]. Zhou et al. prepared Ce-doped MoO_3_ ultrathin nanoflakes with abundant oxygen vacancies by a simple hydrothermal method. The results confirmed that Ce doping not only controls the thickness of nanosheets but also additionally introduces abundant oxygen vacancies, which, together, have a significant effect on the electrochemical performance [6]. Xu et al. synthesized boron-doped NiCo_2_O_4_ with rich oxygen vacancies on carbon fabrics. The introduced B and oxygen vacancies effectively accelerate ion and electron transport and increase the redox reactive sites, which endow the B-NiCo_2_O_4_ electrode with high capacitance and superior rate performance [7]. From the above examples, it is clearly seen that the synergistic effect between doping and oxygen vacancies can enhance the electrochemical properties through the optimization of surface chemistry properties. So far, only a few studies in the literature on MnCo_2_O_4.5_ surface modification through vacancies and doping have been reported for supercapacitors. Therefore, to further understand the role of surface chemistry properties (vacancies and doping) on MnCo_2_O_4.5_ electrochemical behavior and improve its electrochemical performance, it is worthy to explore facile ways to introduce vacancies and doping. 

An ultra-long cycle life and high-power performance are significant advantages of supercapacitors compared to lithium-ion batteries. These superior structural properties are essential for realizing long-cycle stability and high-power output, which are mainly dominated by the following three aspects: First, a robust skeleton can effectively retard or prevent structural aggregation, pulverization, and collapse when electrode materials suffer from stress and strain during the charge/discharge process. Second, a rational porosity and porous structure enable fast ion diffusion and electrolyte penetration, leading to the reduction in polarization and boost rate capability. Third, high surface areas can offer rich electroactive sites, which are required for high capacitance. In various structures, hollow structures hold the potential to realize a long cycle life and the high-rate capacity for supercapacitors in terms of the above criterion, such as a robust skeleton, abundant porosity, and large specific surface area. For instance, MnCo_2_O_4_ hollow spheres on nickel foam were synthesized by Fu’s group in a simple two-step synthetic method, which showed good cycling stability with a capacitance retention of 94% after 10,000 charge–discharge cycles [8]. Zhou and her co-workers used electrospinning technology to prepare one-dimensional ZnMn_2_O_4_ hollow nanofibers for supercapacitors. This electrode possessed long-term cycling stability with a slight upward capacitance (100.8%) after 5000 cycles [9]. To further improve the specific capacitance without sacrificing long cycle life, some researchers constructed complicated structures based on the hollow structure to increase the specific surface area and active sites. In various electrode structures, hollow structures with nanosheets as their secondary structure have been reported for enhanced capacitance. Zhang et al. reported that NiCo-LDH hollow spheres with frizzy NiCo-LDH nanosheets showed a high specific capacitance of 1962 F g^−1^ at 1 A g^−1^ and a good capacitance retention rate of 66.4% at 30 A g^−1^ [10]. The hollow structure can effectively alleviate the serious agglomeration of the LDH outer nanosheet shells. The preserved nanosheets expose more active sites for the redox reactions. Wei et al. prepared Ni-Mn hydroxide hollow spheres with ultrathin nanosheet subunits using an etching strategy, and the unique structure possessed a specific capacity of 1680 F g^−1^ at 2.0 A g^−1^ and impressive cycling stability after 5000 cycles [11]. Li et al. reported hierarchical NiCo_2_O_4_ architectures with 2D/3D nickel foam via a facile one-step way, and 2D nanosheets were in situ generated from the self-evolution of initial NiCo_2_O_4_ nanocages. This 2D/3D hierarchical structure delivered an excellent capacity (1.29 mAh cm^−2^ at 4 mA cm^−2^) and robust cycle stability (113.7% after 5000 cycles) for supercapacitors [12]. Although nanosheets as a secondary structure can significantly enhance the capacitive performance of hollow structures, it still remains a great challenge to find a facile and scalable method to tune the density of nanosheets and further optimize electrochemical performance. Taken together, the exploration of the combination of the surface chemistry and bulk structure is an efficient route to simultaneously enhance energy/power and the cycle lifespan of supercapacitors. 

In this work, hydrangea-like P-MnCoO was successfully prepared for supercapacitors via a facile H_2_O/C_2_H_5_OH-treated and phosphorylated strategy. During the synthesis process, H_2_O/C_2_H_5_OH demonstrates the two birds with one-stone effect: structure-guiding agents for the formation of 2D nanosheets from the self-evolution of initial glycerate nanospheres and the promoting effect and introduction of surface vacancy, which is validated by EPR. In addition, the density or thickness of nanosheets can be well-controlled by simply tuning the volume ratio of ethanol to water, which then affects supercapacitors’ performance. After surface treatment and structural optimization, P-MnCoO possesses desirable physicochemical properties for supercapacitor application as follows: (1) 2D nanosheets provide more accessible active sites to store greater charge; (2) hollow structures not only facilitate both ion and electron transportation and diffusion but also buffer volume change during continuous charge and discharge; (3) the introduced P and oxygen vacancies effectively increases the redox reactive sites and accelerates ion and electron transport, which is confirmed by both experimental results and theoretical calculations. Based on surface modification and structural advantages, P-MnCoO exhibits higher specific capacitance and rate capability than both MnCoO (direct calcination) and H-MnCoO (water treatment and calcination). The assembled asymmetric supercapacitors with P-MnCoO as the positive electrode and activated carbon as the negative electrode show high energy density and superior long-term cycling stability. The facile and low-cost synthesis method may provide the potential for an industrial, scalable application.

## 2. Results and Discussion

### 2.1. Preparation Process

**Figure 1:**
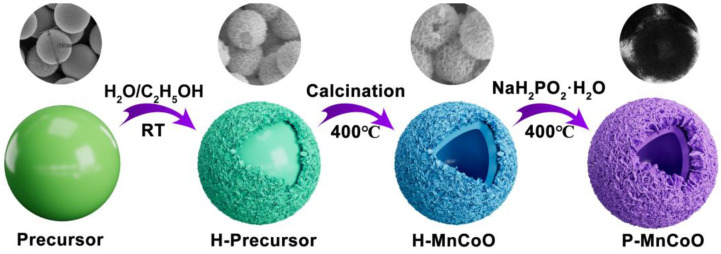
Schematic diagram for preparing P-MnCoO.

The schematic diagram for preparing P-MnCoO is shown in Figure 1. Firstly, monodispersed glycerate nanospheres with a smooth surface were synthesized via the solvothermal method. Secondly, these nanospheres were soaked in a water/ethanol mixed solvent for several hours to prepare the H-Precursor. This process leads to the formation of nanosheets on the surface of these spheres. Subsequently, the solid nanospheres with nanosheets gradually transform into hollow spheres after calcination above 400 °C. Finally, P-MnCoO was synthesized through phosphorization under the same temperature. Representative morphologies of the product at each step are shown above the corresponding structure diagram.

### 2.2. Structure Characterization

**Figure 2:**
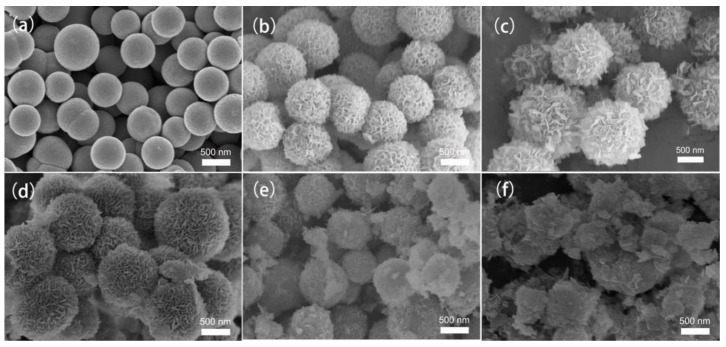
SEM images of H-Precursor with different ratios of ethanol/water treatment for 6 h, (**a**) 1:0, (**b**) 2:1, (**c**) 1:1, (**d**) 1:2, (**e**) 1:3, and (**f**) 0:1.

To reveal the evolution process of the unique structure, morphologies, and phases are further characterized by SEM and XRD. In the solvothermal process, glycerol molecules tend to self-assemble into uniform nanospheres in 2-propanol through strong intermolecular hydrogen bonds and then react with cobalt and manganese to form bimetallic glycerate nanospheres [13]. As shown in Figure S1 in the Supporting Information, solid nanospheres exhibit a smooth surface with an average diameter of about 600 nm. The XRD pattern shows two typical characteristic peaks located at around 11° and 35°, confirming the formation of metal glycerate [14,15,16]. In order to explore the influence of the surface treatment on glycerate nanospheres, we applied water/ethanol treatment with different ratios to monitor the surface change of the materials. From Figure 2a–f, it can be observed that the nanosheets gradually became large and thick with increasing water content in the mixed ethanol/water solvent. When the water/ethanol ratio exceeded two, numerous nanosheets started to stack together and then aggregate into separated clumps or particles. It is known that appropriate nanosheet density is in favor of the electrolyte penetration and exposure of active sites. Hence, materials treated with 1:1 water/ethanol can be assigned as the main representative sample for further research efforts. According to Figure S2, the two sharp peaks at 11.8° and 34.2° are ascribed to MnCo-LDH, which is consistent with the previously published literature [17,18].

**Figure 3:**
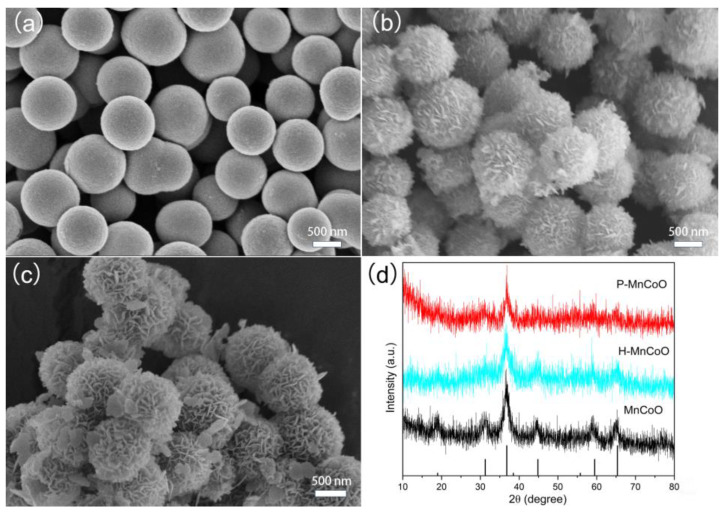
SEM images for (**a**) MnCoO, (**b**) H-MnCoO and (**c**) P-MnCoO; and (**d**) XRD patterns.

After calcination under air, the glycerate nanospheres were converted to MnCo_2_O_4.5_ nanospheres, which was identified by XRD (Figure 3d). In comparison to the glycerate (precursor), the morphology and microstructure of MnCo_2_O_4.5_ can be well preserved (Figure 3a). Similarly, H-MnCoO also retains its nanosheet/nanosphere morphology after water/ethanol treatment followed by calcination and appears almost the same as the water/ethanol-treated sample without calcination, indicating good structural robustness (Figure 3b). Compared with H-MnCoO, which has densely distributed thin nanosheets, P-MnCoO possesses relatively sparse and thick nanosheets after phosphorization. Although scattered/weak adhesive nanosheets detach from the surface of the sphere (Figure 3c), the overall structure does not change significantly. Figure 3d depicts the XRD patterns of MnCoO, H-MnCoO, and P-MnCoO. Clearly, the crystal phase structures of the three samples all correspond to MnCo_2_O_4.5_ (JCPDS card no. 32-0297). In comparison with MnCoO and H-MnCoO, P-MnCoO shows relatively low crystallinity, as evidenced by its weak diffraction peaks and high baseline in the low-angle region, which is in good agreement with the reported literature [19].

**Figure 4:**
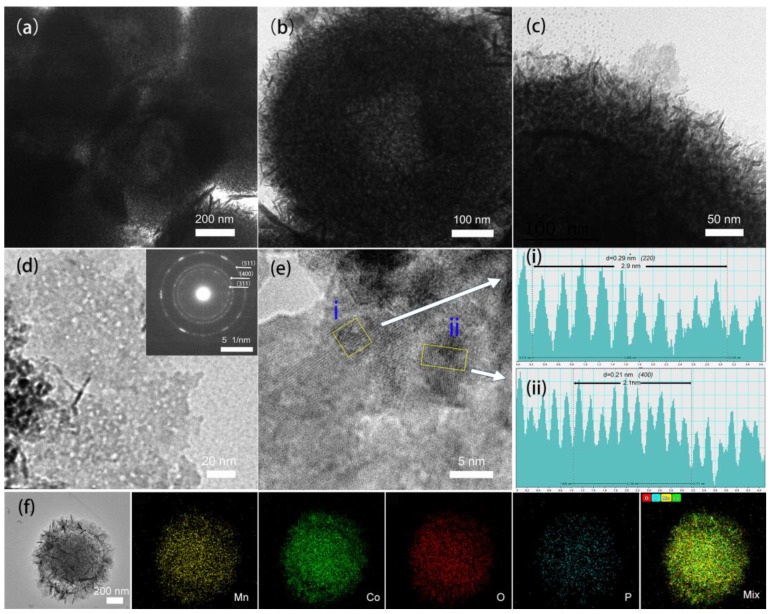
TEM images (**a**–**c**) of P-MnCoO. TEM image (**d**) of P-MnCoO nanosheet detached from the nanosheet–nanosphere structure. The inset is a corresponding SAED image. HRTEM image (**e**) and the corresponding lattice spacing obtained from the line intensity profiles (**i**) and (**ii**). EDS elemental mappings (**f**) of P-MnCoO.

The structure of as-synthesized hydrangea-like P-MnCoO is further elucidated by TEM characterization. As shown in Figure 4a, the well-defined hollow structure in the thin layer area can be clearly visualized, while stacking areas hide hollow morphology. Moreover, a magnified TEM image of a single particle unambiguously reveals that P-MnCoO possesses a unique hydrangea-like structure with an inner hollow core structure and outer nanosheet shells (Figure 4b). The diameter of the hollow core structure is about 100 nm. A closer observation of the shells of P-MnCoO shows that the shell with a thickness of around 50 nm is composed of thin nanosheets (Figure 4c). This hollow core structure with nanosheet shells usually has a large specific surface area, which is helpful in offering abundant active sites to boost electrochemical reactions. As determined by the N_2_ sorption measurement (Figure S3), P-MnCoO possesses a higher BET-specific surface area of 170.1 m^2^ g^−1^ than H-MnCoO (110.9 m^2^ g^−1^) and MnCoO (46.1 m^2^ g^−1^). Compared with MnCoO (inset of Figure S3), P-MnCoO and H-MnCoO exhibit a wider pore size distribution, indicating rich channels, which can efficiently promote ion diffusion. In particular, P-MnCoO contains a micro–meso–macropores construction, while H-MnCoO only consists of a micro–meso construction. The existing macropores structure could act as an ion-buffering reservoir and then facilitate the rapid adsorption of electrolyte ions [20]. It can be intuitively observed that the surface of the nanosheet has lots of holes, as shown in Figure 4d. This morphology mainly originates from the decomposition of glycerate and the etching of PH_3_ during phosphorization [21]. The SAED pattern (inset of Figure 4d) presents a polycrystalline feature with three diffraction ring patterns of (311), (400), and (511), which correspond to the MnCo_2_O_4.5_ phase. Meanwhile, the lattice fringes of 0.29 and 0.21 nm (Figure 4e) are obtained from line intensity profiles, which can be indexed to the (220) and (400) planes of the MnCo_2_O_4.5_ phase (JCPDS card no. 32-0297). The EDX elemental mapping for single particles shown in Figure 4f reveals that the Mn, Co, O, and P elements are homogeneously distributed throughout the whole P-MnCoO domain. The atomic ratio of Mn/Co/O/P is about 1:2.36:3.86:0.19 according to the EDX spectra of P-MnCoO (Figure S4). The deviation from this theoretical value (MnCo_2_O_4.5_) could be ascribed to surface P doping and oxygen vacancy (Ov). The low-resolution SEM image, including several particles and corresponding EDX elemental mappings (Figure S5), further verifies the homogeneous distribution of Mn, Co, O, and P elements in the whole area.

**Figure 5:**
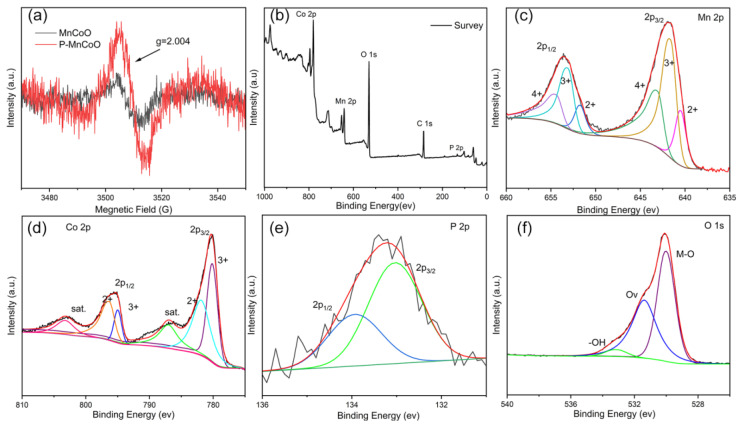
EPR spectra of P-MnCoO and MnCoO (**a**), XPS spectrum of the survey (**b**), Mn 2p (**c**), Co 2p (**d**), P 2p (**e**), and O 1 s (**f**).

Electron paramagnetic resonance technology (EPR) and X-ray photoelectron spectroscopy (XPS) were used to further examine surface electronic states. As displayed in Figure 5a, both P-MnCoO and MnCoO have a similar EPR signal at g = 2.004, which is correlated with the generation of Ov. Significantly, the EPR signal intensity of P-MnCoO is remarkably stronger than that of MnCoO, indicating the increase in Ov after phosphorization. In the XPS survey spectra of P-MnCoO (Figure 5b), it can be seen that Mn, Co, P, and O elements coexisted in the material. Figure 5c is a high-resolution XPS spectrum of Mn 2p and contains two major peaks at the binding energies of 641.9 eV and 653.6 eV, which correspond to Mn 2p1/2 and Mn 2p3/2, respectively. Moreover, the XPS spectrum of the Mn 2p region can be divided into four peaks through refined fitting. The fitting peaks located at 640.5 eV and 651.8 eV are assigned to the Mn 2p3/2 and Mn 2p1/2 of Mn^2+^ ions, respectively, while the peaks at 641.8 eV and 653.3 eV are ascribed to the Mn 2p3/2 and Mn 2p1/2 of Mn^3+^. Moreover, the peaks centered at 643.3 eV and 654.7 eV are assigned to Mn^4+^. In the Co 2p XPS spectra shown in Figure 5d, two pairs of dominant peaks correspond to Co 2p1/2 and Co 2p3/2, respectively. The fitted peaks appear at 780.2 eV and 795.0 eV belong to Co^3+^, and the peaks at 781.9 eV and 796.6 eV are attributed to Co^2+^. In addition, two broad peaks at 803.2 eV and 987.1 eV are the corresponding satellite peaks of Co 2p. In the 2p XPS spectra, the two peaks at 133.1 and 133.9 eV can be ascribed to metal phosphide [22,23]. The XPS spectrum of O 1s consists of three peaks at 530.1, 531.4, and 533.1 eV, which originated from metal–oxygen, oxygen deficiency, and hydroxyl oxygen. The above results prove the coexistence of surface Ov and phosphate-ion modulation. According to the data of XPS, the atomic ratio of Mo/Co/O/P is 1:1.97:5.56:0.29. Compared with the previous EDX results, the surface contents of O and P exceed those of the bulk structure, further confirming the effect of surface composition during phosphorization.

### 2.3. Electrochemical Performance

**Figure 6:**
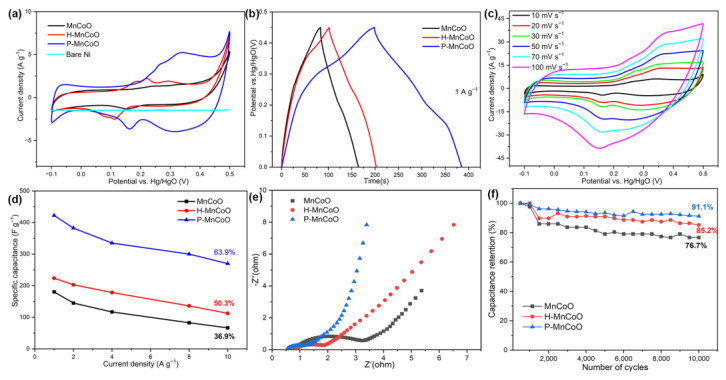
(**a**) CV curves of the MnCoO, H-MnCoO and P-MnCoO electrode at 10 mV/s, (**b**) GCD curves of three electrodes at 1 A/g, (**c**) CV curves of the P-MnCoO electrode at various scan rates, (**d**) rate performance of the three electrodes from 1 A/g to 10 A/g, (**e**) EIS Nyquist plots of three electrodes, and (**f**) cycling performance of three electrodes at 10A/g.

To examine the charge storage performance of as-fabricated P-MnCoO for the supercapacitor, CV and GCD tests of the three-electrode system were performed to measure electrochemical performance using the 6 M KOH solution as the electrolyte. Figure 6a shows the CV curves of MnCoO, H-MnCoO, and P-MnCoO at the scan rate of 10 mV/s. It is clear that the enclosed CV curve area of the P-MnCoO is apparently larger than those of the pure Ni foam, MnCoO, and H-MnCoO, indicating that P-MnCoO has a larger specific capacitance than the other materials. This intuitive capacitive behavior can be further verified by the GCD curves. As shown in Figure 6b, P-MnCoO exhibits a much longer discharging time in comparison to the other two materials at the current density of 1 A/g. Compared with MnCoO and H-MnCoO, the results prove that the Ov and hollow structure with 2D nanosheets on the surface of P-MnCoO can significantly improve the capacitive performance, which may contribute to enhanced surface reactivity and increased active sites. Figure 6c shows the CV curves of the P-MnCoO electrode at various scan rates. All CV curves have similar shapes within two pairs of redox peaks (Co^2+^/Co^3+^ and Mn^2+^/Mn^3+^) [24], confirming the pseudocapacitive characteristics. A similar phenomenon is also observed for both the MnCoO (Figure S6a) and H-MnCoO electrode (Figure S6b), and both show pseudocapacitive behavior due to faradaic redox reactions. Rate capability is an important parameter in estimating the power applications for supercapacitors. The GCD curves of MnCoO, H-MnCoO, and P-MnCoO electrodes at various current densities are shown in Figure S7a–c, respectively. The corresponding specific capacitance can be calculated according to formula (1), which is plotted in Figure 6d. Specifically, the P-MnCoO electrode delivers a higher capacitance (425 F/g) than that of the MnCoO electrode (180 F/g) and H-MnCoO electrode (225 F/g) at a current density of 1 A/g. When the current density increases from 1.0 to 10 A/g, the discharge capacity of P-MnCoO decreases from 425 to 271.5 F/g with a capacity retention of 63.9%, which is higher than that of the MnCoO (36.9%) and H-MnCoO (50.3%) electrode. EIS techniques can be used to investigate the insights into the advantages of electronic/ion transfer and the reaction kinetics of these electrodes. In Figure 6e, all Nyquist plots consist of three regions according to the different frequency ranges corresponding to the different interfacial processes. Briefly, the high-frequency intercept on the real Z’ axis represents the series resistance (Rs). In the middle-frequency region, the semicircle can be attributed to the charge transfer resistance (Rct) on the electrode surface [25,26]. The P-MnCoO electrode has a much smaller intercept and semicircle diameter than the H-MnCoO or MnCoO electrode, indicating a lower Rs and Rct of the P-MnCoO electrode, which is caused by Ov and phosphate-ion modulation. The straight line in low frequency is associated with the ion diffusion of the electrolyte. The steeper slope of straight lines at the low frequency of the P-MnCoO electrode demonstrated superior electrolyte ionic diffusion behavior, and this can be mainly attributed to the hollow structure and 2D nanosheets offering abundant ion transmission channels [27,28,29]. Therefore, the improved conductivity and mass transport of the P-MnCoO electrode enhances its electrochemical performance. A good hydrophilic surface can facilitate electrolyte penetration. The P-MnCoO electrode displays a small contact angle (Figure S8), indicating relatively high wettability. Compared with MnCoO, the wettability of P-MnCoO is significantly increased, which originates from surface modification (Ov and phosphorization). Moreover, the P-MnCoO electrode shows outstanding long-term cycling stability with 91.1% retention after 10,000 concomitant cycles, which is superior to that of H-MnCoO (85.2%) and MnCoO (76.7%). In addition, the performance of the P-MnCoO electrode is much superior to previously reported results with respect to specific capacitance and cycle lifetime, as summarized in Table S1 [30,31,32,33,34]. Compared with similar materials reported earlier, the enhanced electrochemical performance of P-MnCoO is mainly due to surface chemistry properties and structural properties. The hollow structure with 2D nanosheets possesses a high specific surface area and abundant pore structure, which can expose more active sites and shorten the ion diffusion distance. The surface chemistry modulation enables P-MnCoO good electronic conductivity and electrolyte wettability.

**Figure 7:**
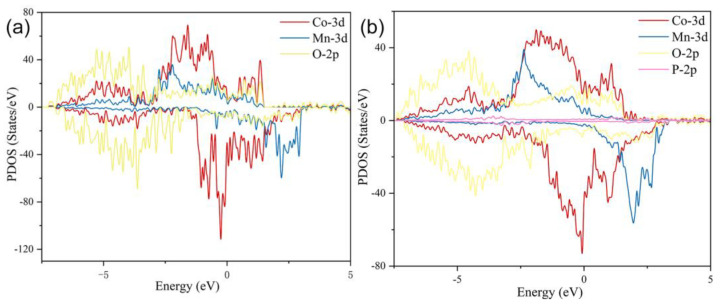
Partial density of states (PDOS) MnCoO (**a**) and P-MnCoO (**b**).

Density functional theory (DFT) calculations have been carried out to further study the effects of oxygen defects and phosphorization on P-MnCoO conductive behavior. The structural models of MnCoO and P-MnCoO are displayed in Figure S9. The calculated PDOS of MnCoO and P-MnCoO are shown in Figure 7a,b. Compared with MnCoO, P-MnCoO possesses a higher charge density near the Fermi level, which can provide more charge carriers for the redox reaction and improve electronic conductivity for charge transportation. 

### 2.4. Applications of Asymmetric Supercapacitor

**Figure 8:**
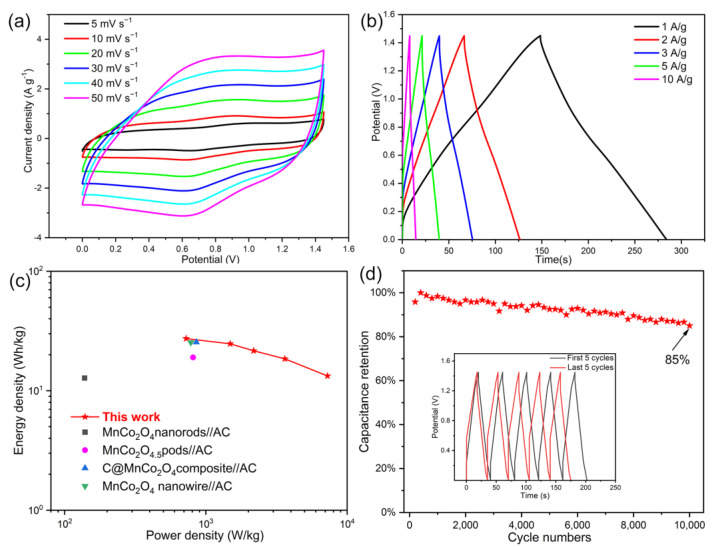
(**a**) CV curves of the device at various scan rates. (**b**) GCD curves of the device at different current densities. (**c**) Ragone plots of the device compared with previously reported data. (**d**) Cyclic stability of the device at 5 A/g. (Inset: GCD curves of the first 5 cycles and the last 5 cycles).

The electrochemical asymmetric supercapacitor (ASC) device is fabricated by assembling as-prepared P-MnCoO as the positive and activated carbon as the negative to evaluate the practical application. According to the charge balance principle between the positive and negative (Q^+^ = Q^+^) based on the capacitances of the two above electrodes (Figure S10), the optimal mass ratio of P-MnCoO to AC is calculated to be 0.42 in the ASC. Figure 8a shows the CV curves of a device at a range of 5–50 mV s^−1^. The device exhibits nearly rectangular-like shapes with distinct redox peaks, suggesting the coexistence of EDLC and pseudocapacitance. Additionally, all the CV curves remain rectangular-like in shape with an increasing sweeping rate, and no clear distortion was observed even at 50 mV s^−1^, demonstrating superior reversibility with fast charge propagation. The GCD curves collected at different current densities from 1 to 10 A/g are shown in Figure 8b. When the current density is 1 A/g, a high specific capacitance of 93.5 F/g is obtained. The specific capacitance still maintains up to 45.5 F/g with 48.7% capacitance retention, indicating its good rate capability. The energy and power densities from the GCD curves at various current densities are shown in the Ragone plot (Figure 8c). This device possesses a high energy density of 27.3 Wh/kg at a power density of 724.9 W/kg and an energy density of 13.3 Wh/kg at a high-power density of 7257.3 W/kg. The achieved performance is superior to or comparable to previously reported metal oxide-based ASC [26,27,28,29]. The cycling life of this device is further evaluated by means of 10,000 charge/discharge cycles at 5 A/g, and the result is displayed in Figure 8d. After 10,000 charge/discharge cycles, 85% of the initial capacitance of the device was retained, revealing its excellent cycling stability. Furthermore, the similar shape between the first and last five cycles of the GCD curve in the inset of Figure 8d also verifies cyclic stability.

## 3. Materials and Methods

### 3.1. Chemicals

Cobalt nitrate hexahydrate (Co(NO_3_)_2_·6H_2_O), manganese nitrate tetrahydrate (Mn(NO_3_)_2_·4H_2_O), glycerol (C_3_H_8_O_3_), isopropanol (C_3_H_8_O), and ethanol (C_2_H_5_OH) were purchased from Sinopharm Chemical Reagent Co., Ltd., Shanghai, China. Sodium hypophosphite monohydrate (NaH_2_PO_2_ H_2_O) was purchased from Aladdin Reagent Co., Ltd., Shanghai, China. Deionized water (DW) was prepared in our laboratory using an ultrapure water machine. All the chemicals in the work were directly used without any further purification.

### 3.2. Synthesis of the MnCoO, H-MnCoO, P-MnCoO Hollow Spheres

The precursor of MnCoO was synthesized based on a previously reported method with some modifications [13]. In a typical procedure, 0.25 mmol Mn(NO_3_)_2_·4H_2_O and 0.5 mmol Co(NO_3_)_2_·6H_2_O were first dissolved in 40 mL of isopropanol. Next, 8 mL of glycerol was added to the solution with vigorous magnetic stirring for 30 min. Then, the as-prepared mixture solution was transferred into a 100 mL Teflon-lined stainless-steel autoclave and heated at 180 °C for 16 h in an electric oven. After cooling to room temperature naturally, MnCoO precursor powders were gained via ethanol washing and drying in an oven at 60 °C. To obtain MnCoO hollow spheres, the above precursor powders were calcined in the air at 400 °C for 2 h with a heating rate of 2 °C min^−1^.

The conversion of the MnCoO precursor to H-MnCoO nanosheet-hollow spheres through water–ethanol treatment was conducted as follows: 100 mg of the MnCoO precursor was dispersed or treated in a 100 mL mixed ethanol/water solvent with various volume ratios, including 1:0, 3:1, 2:1, 1:1, 1:2, 0:1, and stirred under 300 r/min for 6 h. After the completion of the reaction, the obtained sample was washed with ethanol several times and then dried overnight in an oven at 60 °C. To obtain H-MnCoO nanosheet-hollow spheres, the ethanol/water-treated product was heated to 400 °C at a ramp rate of 2 °C min^−1^ in an air atmosphere and maintained for 2 h followed by natural cooling. P-MnCoO nanosheet-hollow spheres were prepared based on H-MnCoO nanosheet-hollow spheres. The as-prepared H-MnCoO powder was placed at the center of a quartz tube furnace, while NaH_2_PO_2_⋅H_2_O powder was placed on the upstream side of the furnace. The mass ratio of H-MnCoO and NaH_2_PO_2_⋅H_2_O was 2. Then, the furnace was heated to 400 °C for 1 h under an argon atmosphere, followed by natural cooling. P-MnCoO nanosheet-hollow spheres were rinsed with ethanol and dried in a vacuum at 60 °C. 

### 3.3. Characterization and Measurements

The phases of the samples were examined by X-ray diffraction (D8 advance, Bruker, Mannheim, Germany). The morphologies were observed by scanning electron microscope energy-dispersive X-ray microanalysis (SEM, Hitachi, SU-70, Tokyo, Japan). The microstructure was observed by transmission electron microscopy (TEM Tecnai F20, Hillsboro, OR, USA). The pore size distributions and specific surface area (SSA) were analyzed by the Micromeritics ASAP 2460 BET apparatus (Micromeritics ASAP 2460, Norcross, GA, USA). Electron spin resonance (ESR) spectra were obtained using the Bruker EMXplus-6/1 (Germany) electron spin resonance spectrometer. The chemical states were identified by X-ray photoelectron spectroscopy (XPS, Thermo Scientific, Waltham, MA, USA).

The powder was used to fabricate the electrode according to the following procedure. Typically, powder (MnCoO, H-MnCoO, or P-MnCoO), carbon black, and PTFE (weight ratio of 8:1:1) with ethanol as the solvent were ground into a homogeneous slurry. The slurry was coated on nickel foam, dried at 110 °C for 12 h, and then compressed at a pressure of 10 MPa for 1 min to obtain the working electrode. The electrochemical properties were evaluated on a CHI 760E electrochemical Working Station and CT2001A LAND test system, including cyclic voltammetry (CV), galvanostatic charge–discharge (GCD), electrochemical impedance spectroscopy (EIS), and cycle life. In a three-electrode cell, the Hg/HgO electrode and Pt plate were used as the reference and counter electrode, respectively, while the 3 M KOH aqueous solution served as the electrolyte. In the two-electrode system, the P-MnCoO and the activated carbon were employed as the positive electrode and the negative electrode, respectively. 

The specific capacity (*C_s_*) was obtained from GCD curves and can be calculated according to the following formula:

(1) $$C_{s}=\frac{I\times ∆t}{m∆V}$$

Here, *C_s_* (F g^−1^), *I* (A), Δ*t* (s), *m* (g), and Δ*V* (V) stand for specific capacitance, current, discharge time, the mass loading of active materials, and voltage change. 

The energy density (E, Wh kg^−1^) and the power density (*P*, W kg^−1^) were obtained in relation to the following equations:

(2) $$E=\frac{1}{7.2}{CV}^{2}$$

(3) $$P=\frac{3600E}{∆t}$$

### 3.4. Theoretical Calculations

We used the DFT, as implemented in the Vienna Ab initio simulation package (VASP), in all calculations. The exchange–correlation potential is described using the generalized gradient approximation of Perdew–Burke–Ernzerhof (GGA-PBE). The projector augmented wave (PAW) method was employed to treat interactions between the ion cores and valence electrons. The plane-wave cutoff energy was fixed to 400 eV. Given structural models were relaxed until the Hellmann–Feynman forces were smaller than −0.05 eV/Å and a change in energy smaller than 10^−5^ eV was attained.

## 4. Conclusions

In summary, we designed and successfully prepared P-MnCoO by considering the effect of surface chemistry and bulk structure on energy storage. The Ov and phosphorization and surface chemical modification can improve electronic conductivity and electrolyte wettability for rapid ion/electron transfer. The hollow hydrangea-like structure with an ingenious construction can effectively provide a large number of active sites, abundant pore structure, and favorable structural stability. Owing to the synergistic effect of surface chemistry and bulk structure, the P-MnCoO electrode exhibited enhanced electrochemical performance. The fabricated P-MnCoO/AC asymmetric supercapacitor delivered a high energy density of 27.3 Wh/kg at a power density of 724.9 W/kg and good cyclability with a capacitance retention of 85.0% after 10,000 cycles.

## Data Availability

Data are contained within the article, further inquiries can be directed to the corresponding author.

## Supplementary Materials

The following supporting information can be downloaded at: https://www.mdpi.com/article/10.3390/ijms25105075/s1.
