# Supplementary material for: Synergy of Oxygen Vacancy and Surface Modulation Endows Hollow Hydrangea-like MnCo2O4.5 with Enhanced Capacitive Performance
Source: Int J Mol Sci. 2024 May 7;25(10):5075. doi: 10.3390/ijms25105075 (PMC11121676; doi:10.3390/ijms25105075)
Supplement: Supplementary file 1 [file ijms-25-05075-s001.zip › ijms-2952531-supplementary.pdf]

Synergy of Oxygen-Vacancy and Surface Modulation Endows hollow hydrangea-like $MnCo_2O_{4.5}$ with Exceptional capacitive performance

Gaofeng Li [1], Yanyan Li [1], Pengfei Wang [2], Lingling Chen [2], Longfei Li [2], Chen Bao [1], Jianfei Tu [1,] and Dianbo Ruan [1,*]

[1] Institute of Advanced Energy Storage Technology and Equipment, Faculty of Mechanical Engineering and Mechanics, Ningbo University, Ningbo, 315211, People's Republic of China; ligaofeng@nbu.edu.cn

[2] Institute of Advanced Energy Storage Technology and Equipment, School of Materials Science and Chemical Engineering, Ningbo University, Ningbo, 315211, People's Republic of China

* Correspondence: ruandianbo@nbu.edu.cn (D.R.); Tel.: 0574-87608848

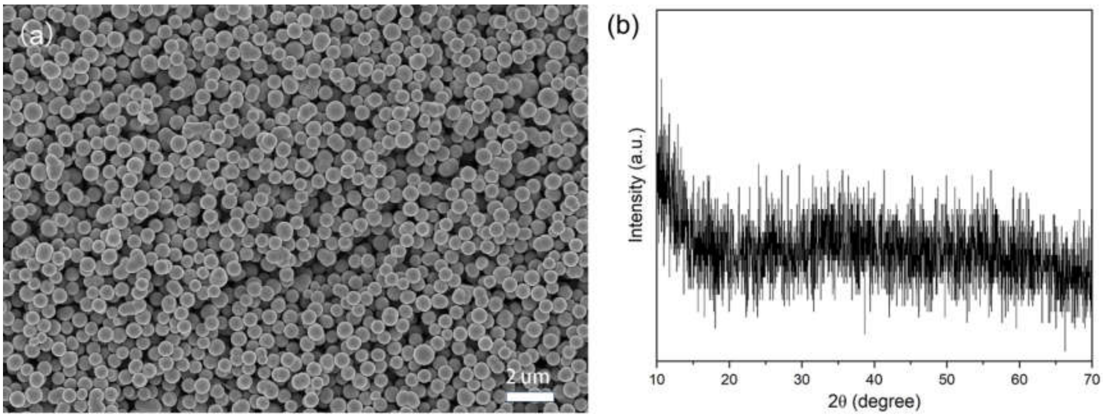

Figure S1. SEM (a) and XRD (b) image of MnCo glycerate solid sphere.

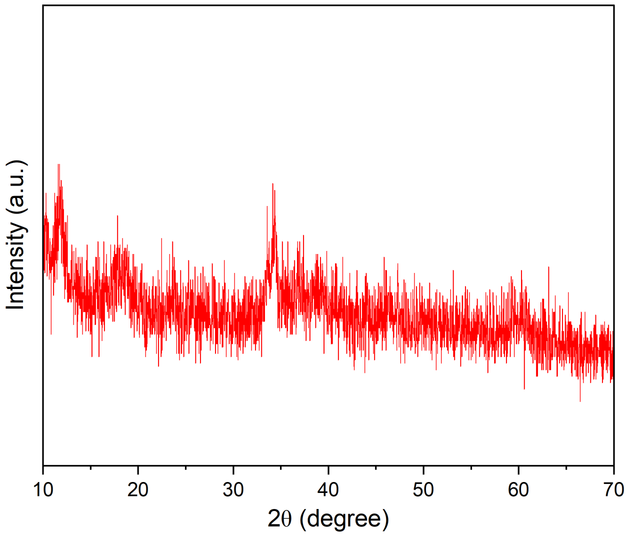

Figure S2. The XRD pattern of H-precursor treated by ethanol/water mixed solvent (1:1).

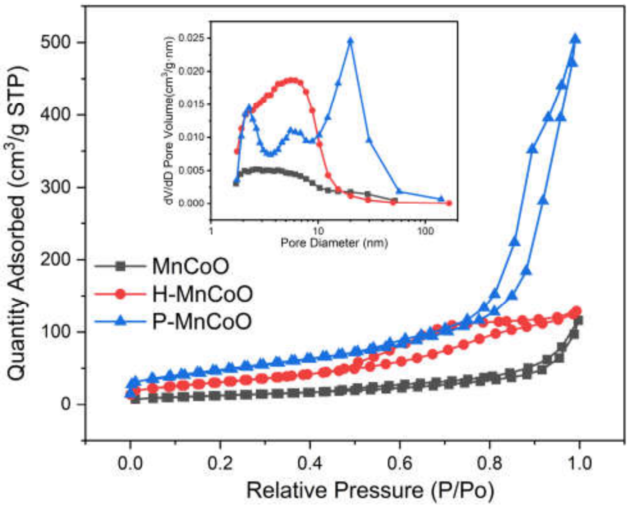

Figure S3. Nitrogen adsorption desorption isotherms of three samples, inset is corresponding BJH plots.

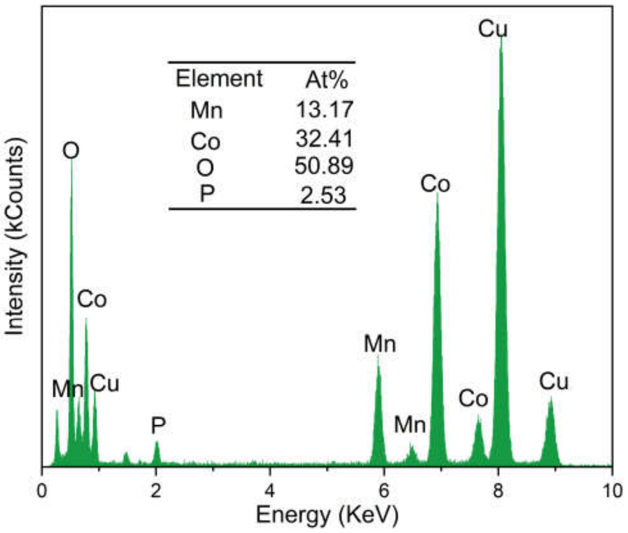

Element	At%
Mn	13.17
Co	32.41
O	50.89
P	2.53

Figure S4. EDX spectra of P-MnCoO material.

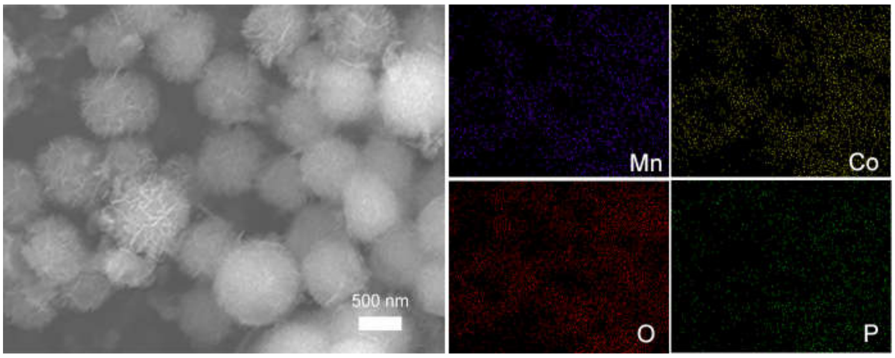

Figure S5. SEM image and corresponding EDX elemental mappings within several particles of P-MnCoO.

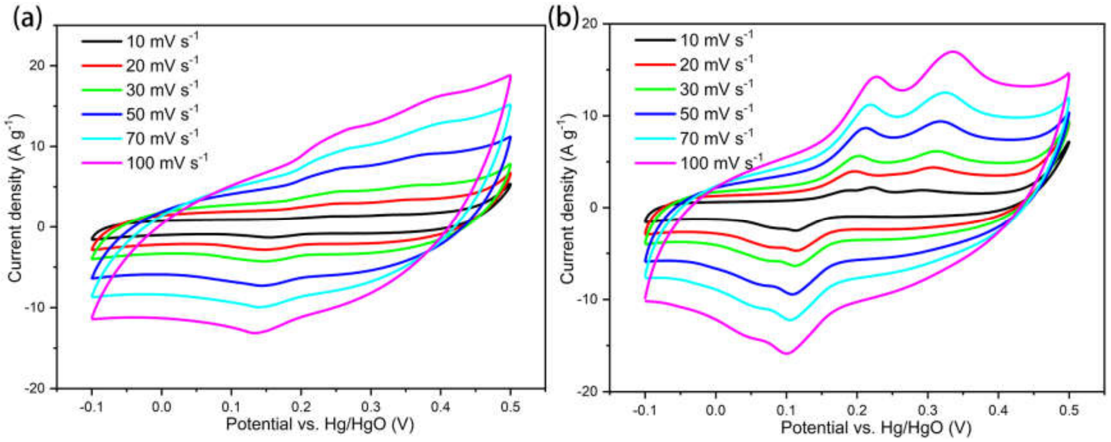

Figure S6. CV curves of (a) MnCoO and (b) H-MnCoO at various scan rates.

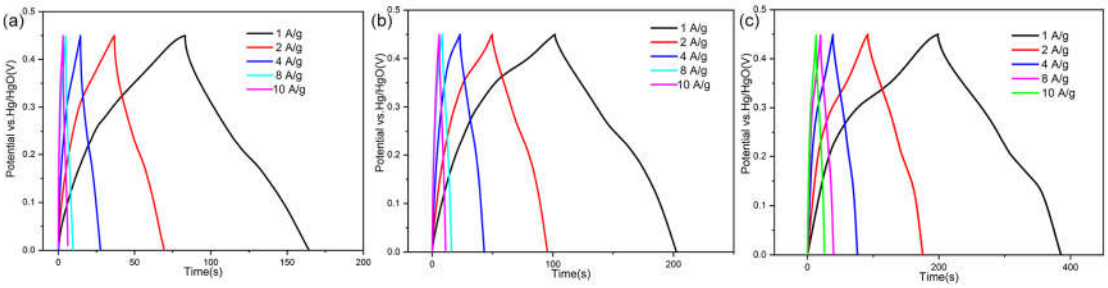

Figure S7. GCD curves of (a) MnCoO, (b) H-MnCoO and (c) P-MnCoO at different current densities.

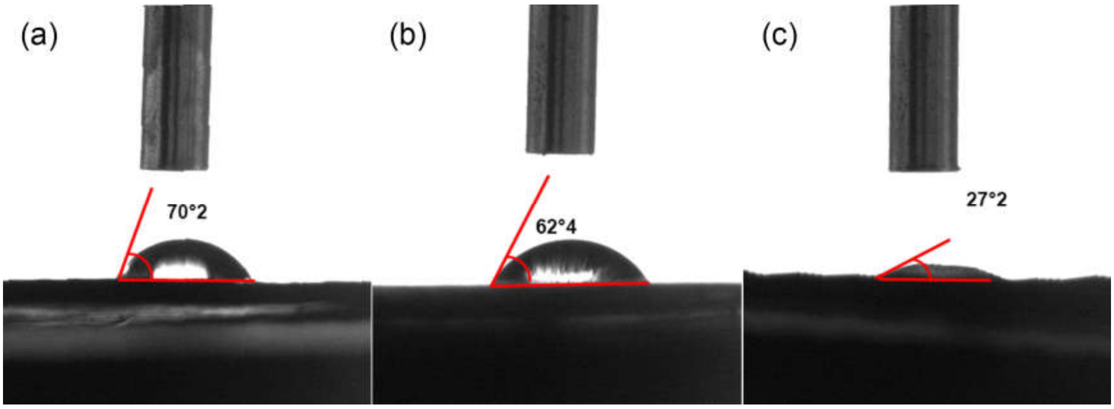

Figure S8. Water contact angle images of (a) MnCoO, (b) H-MnCoO and (c) P-MnCoO electrodes.

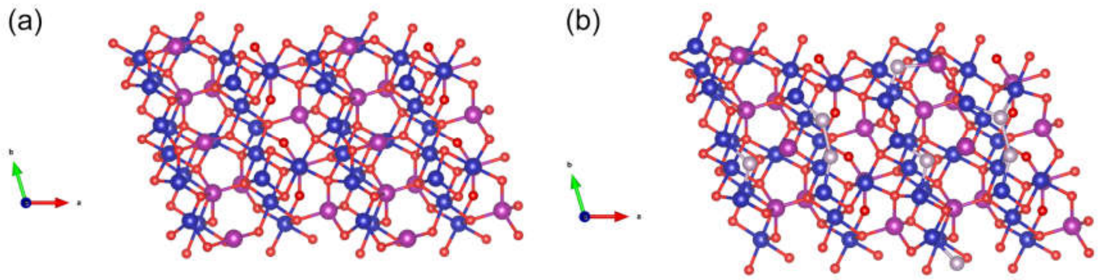

Figure S9. The crystallographic models of (a) MnCoO and (b) P-MnCoO electrodes.

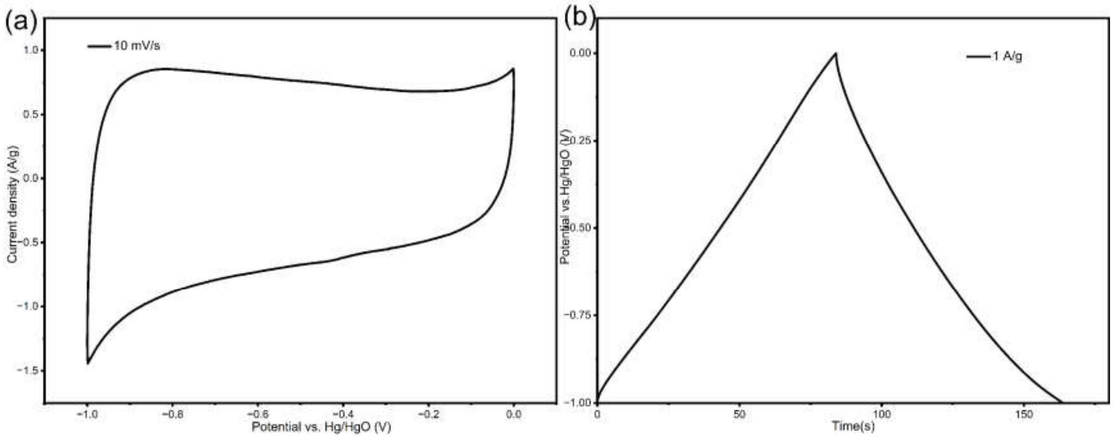

Figure S10. CV (a) and GCD curves (b) of AC electrode.

Table S1

Various pseudocapacitive electrodes in supercapacitors.

Electrode materials	Current density(A g^{-1})	Capacitance (F g^{-1})	Retention (Cycles)	Ref
$MnCo_2O_4$ hollow microspheres	1	235.7	93.6 % (2000)	[S1]
$MnCo_2O_{4.5}$ spindle-like microstructures	0.5	343	81.3 % (5000)	[S2]
$MnCo_2O_{4.5}$/graphene composite	0.5	252.3	92.6 %(1000)	[S3]
$MnCo_2O_{4.5}$ nanowire	1	405	91 %/(3000)	[S4]
$MnCo_2O_{4.5}$@$NiCo_2O_4$ nanowire composite	1	325	70.5 %(3000)	[S5]
P-MnCoO hollow hydrangea-like structure	1	425	91.1 %(10000)	**This work**

References

1. Che, H.; Liu, A.; Mu, J.; Wu, C.; Zhang, X. Template-free synthesis of novel flower-like $MnCo_2O_4$ hollow microspheres for application in supercapacitors. *Ceram. Int.* **2016**, *42*, 2416-2424. 10.1016/j.ceramint.2015.10.041
2. Liao, F.; Han, X.; Zhang, Y.; Xu, C.; Chen, H. Solvothermal synthesis of porous $MnCo_2O_{4.5}$ spindle-like microstructures as high-performance electrode materials for supercapacitors.

Ceram. Int. **2018**, *44*, 22622-22631. 10.1016/j.ceramint.2018.09.038

3. Li, Y.; Peng, X.; Xiang, J.; Yang, J. Synthesis of $MnCo_2O_{4.5}$/graphene composite as electrode material for supercapacitors. *Int. J. Electrochem. Sci.* **2017**, *12*, 10763-10772. 10772.10.20964/2017.11.63
4. Li, S.; Wang, J.; Wang, M.; Ni, Y. $MnCo_2O_{4+x}$ nanowire arrays grown on carbon sponge: improved electrochemical and catalytic performances. *CrystEngComm*, **2019**, *21*, 403-410. 10.1039/C8CE01793J
5. Facile synthesis of manganese cobalt oxide/nickel cobalt oxide composites for high-performance supercapacitors. *Front. Chem.* **2019**, 6, 661. 10.3389/fchem.2018.00661
